# Dynamic 3D Scene Depth Reconstruction via Optical Flow Field Rectification

**DOI:** 10.1371/journal.pone.0047041

**Published:** 2012-11-09

**Authors:** You Yang, Qiong Liu, Rongrong Ji, Yue Gao

**Affiliations:** 1 Department of Electronics and Information Engineering, Huazhong University of Science and Technology, Wuhan, Hubei, China; 2 Department of Automation, Tsinghua University, Beijing, China; 3 Department of Electrical Engineering, Columbia University, New York, New York, United States of America; 4 School of Computing, National University of Singapore, Singapore, Singapore; National Microelectronics Center, Spain

## Abstract

In this paper, we propose a depth propagation scheme based on optical flow field rectification towards more accurate depth reconstruction. In depth reconstruction, the occlusions and low-textural regions easily result in optical flow field errors, which lead ambiguous depth value or holes without depth in the obtained depth map. In this work, a scheme is proposed to improve the precision of depth propagation and the quality of depth reconstruction for dynamic scene. The proposed scheme first adaptively detects the occlusive or low-textural regions, and the obtained vectors in optical flow field are rectified properly. Subsequently, we process the occluded and ambiguous vectors for more precise depth propagation. We further leverage the boundary enhancement filters as a post-processing to sharpen the object boundaries in obtained depth maps for better quality. Quantitative evaluations show that the proposed scheme can reconstruct depth map with higher accuracy and better quality compared with the state-of-the-art methods.

## Introduction

Depth maps are crucial for three-dimensional (3D) imaging [1] and displaying, and have been widely used in digital holography image processing [2], [3], object reconstruction in integral imaging [4], [5], 3D object retrieval [6]–[10] and tomographic phase microscopy [11]. Practically, high fidelity depth maps of a dynamic scene are calculated or captured in a temporal discrete manner due to the intensive computational complexity of depth reconstruction. For example, the widely used RGB-D [12], [13] (e.g., Kinect) and ToF [14], [15] camera can capture the depth map in video-rate but with low resolution (e.g., 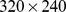 pixels). The devices have challenges to capture depth maps for dynamic scene in video-rate with higher resolution (e.g., standard definition or even higher). In many 3D applications, it is noted that higher capture-rate depth map sequence with higher resolution is required to better represent a dynamic scene [13], [15]–[18].

In order to solve the problem, depth propagation algorithms [19]–[21] have been investigated to compensate the capture-rate to video-rate of depth maps in recent years. In these algorithms, it is assumed that the variation for a given dynamic scene is identical for both the depth and the color information of one viewpoint. Specifically, objects keep static in consequent color frames will not arouse depth value variation for these objects, and the depth value for the region containing static object also keeps static in depth map. On the other hand, motions in consequent color frames correspond to depth value variation in the same region. Therefore, the status (i.e., static or motive) of object in consequent color frames can be used to describe the depth value variation in depth map. Motion vector is widely applied to describe the motion status of objects, and it can be obtained with pixel-, block- or region-wise and with different accuracy. For the case of depth propagation, the pixel-wise motion vectors (PMVs) in consequent color frames with high accuracy can be mapped to depth maps also with high accuracy. Based on the assumption, low capture-rate depth maps can be compensated to video-rate. In this case, the captured and high resolution depth maps can be treated as key frames, and the depth information in to-be-reconstructed depth maps is propagated from the key frame by the obtained PMVs.

The main problem for depth propagation is that it is very challenging to obtain accurate PMVs for the occlusive or low textural regions, although PMV can have high accuracy in other regions. Inaccurate PMVs in these regions may lead to ambiguities and holes in the reconstructed depth maps, which decrease the reconstruction quality significantly. Variety of methods were proposed to improve the quality of reconstructed depth maps. For example, manual marking from users on potential problem regions can improve the quality of reconstructed depth maps significantly [20], but this method is not applicable in many automatic processing systems. Some filters have been applied in post-processing of reconstructed depth map, such as bilateral filter [19], [21], discontinuity analysis and interpolation [22] and in-painting [23]. These filters usually result in inevitable and undesired blurs for depth maps, and these artifacts are unfavorable for 3D dynamic scene representation. In order to solve this problem, we propose to rectify the optical flow field before propagation rather than rectify the depth results after propagation. The quality of the reconstructed depth is improved by PMVs rectification since global filtering has been avoided in this method. Furthermore, a boundary enhancement filter is proposed to refine the edges of the reconstructed depth maps. The main contributions of our work are three-fold: (1) propose a depth propagation scheme based on optical flow field rectification, in which high accurate PMVs can be obtained to improve the precision of propagation and the quality of reconstructed depth map, (2) propose an adaptive occlusive and low textural regions detection and rectification method for PMVs, and (3) propose a boundary enhancement filter to refine the reconstructed depth map.

## Materials and Methods

### Overview of the Proposed Rectification Method

In this work, we reconstruct depth maps for dynamic 3D scene in video-rate by propagation. The PMVs among consequent color images describe the temporal correlations in pixel-wise, and can be applied in propagation from the key depth map to the consequent vacant depth maps. Therefore, the quality of reconstructed vacant depth map highly depends on the precision of obtained PMV. Originally, PMVs can be calculated by traditional optical flow algorithms or motion estimation methods. However, the precision of obtained PMV decreases in several regions, for example, the occlusive or low textural regions. In these regions, less information is available to the matching procedure in determining PMV, and thus errors in obtained PMV are inevitable. These errors will result in unreliable PMVs for depth reconstruction. In order to improve the quality of reconstructed depth maps, the obtained PMVs are rectified before propagation and reconstruction in our work.

**Figure 1:**
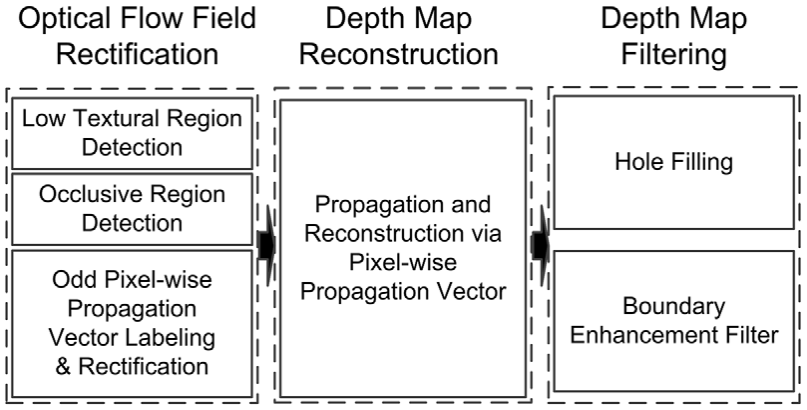
The schematic overview of our method.

Figure 1 shows the schematic overview of our proposed scheme. As we mentioned above, a rectification on PMVs is performed after PMVs have been obtained by optical flow algorithm. The rectification is performed to solve the problem caused by the occlusive and low textural regions. After that, depth information in key depth map is propagated to vacant depth maps through the rectified PMVs for depth map reconstruction. Finally, a depth map filtering will be performed finally to improve the quality of reconstructed depth. The details of each step of our proposed scheme will be given in following subsections.

### Optical Flow Field Rectification

The unreliable PMVs usually occur in occlusive or low textural regions as aforementioned. Therefore, these regions should be detected properly at first.

The texture complication is an important clue for the goal of detection. Usually, the texture complication keeps consistency with the variation of pixel value, so that it can be represented by standard deviation of pixel values. The Heaviside step function is a unit step function, and it can be denoted by
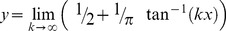
(1)


**Figure 2:**
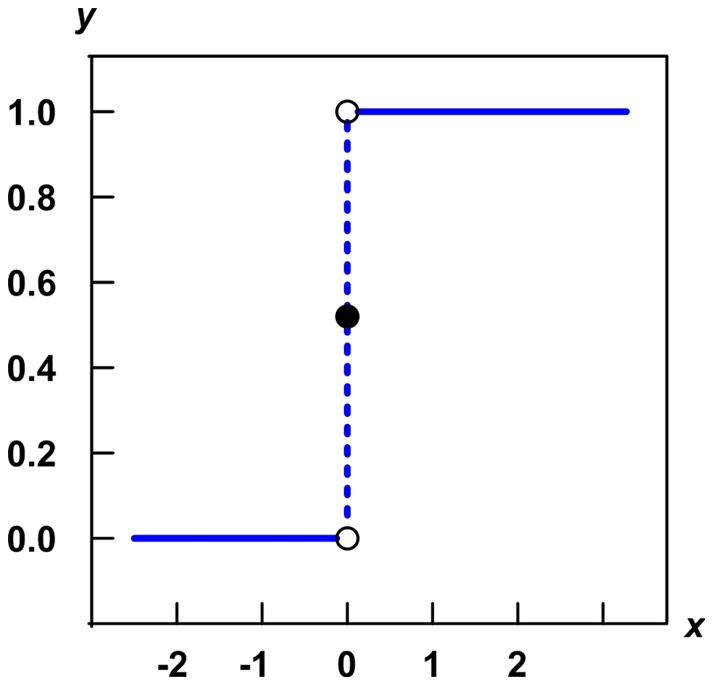
Heaviside step function.



This function is always applied in the mathematics of control theory and signal processing. This function is a discontinuous function whose value is 0 for negative argument and 1 for positive argument. As shown by Figure 2, the function represents a signal that switches on at a specified time (usually triggered by a threshold) and stays switched on indefinitely. In order to differentiate the pixel 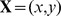 in color image, we propose a binary decision function 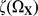 in form of the Heaviside step function to determine whether a region 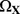 is the low textural region by

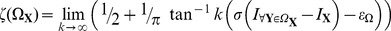
(2)where 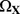 is the neighboring pixel set centered at 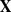, 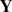 is a pixel in 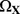, 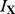 is the gray value for 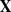, 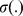 is standard deviation operator for a set, and 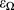 is a threshold for texture. According to the definition of Heaviside step function, the value of 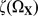 corresponds to a binary decision for textural region detection. 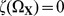 indicates the pixel 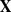 is surrounded by textures, but in low textural region when 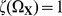.

Similarly, we also propose a binary decision function 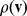 to determine whether a pixel 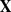 is occluded
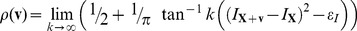
(3)where 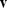 is the PMV on 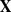, 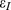 is a threshold for occlusions. 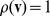 is for the occluded pixel 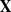, and 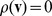 is for the visible one. In determining the occlusive or low textural regions, a smaller threshold is related to accurate decisions and stable performances on different test materials, while increase the computation complexity and unfavorable for implementations. On the other hand, larger threshold is benefit for implementations, but decrease the accuracy of decision and have unstable performances. We will discuss the parameter settings for thresholds 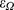 and 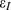 in the section of experiments in details.

**Figure 3:**
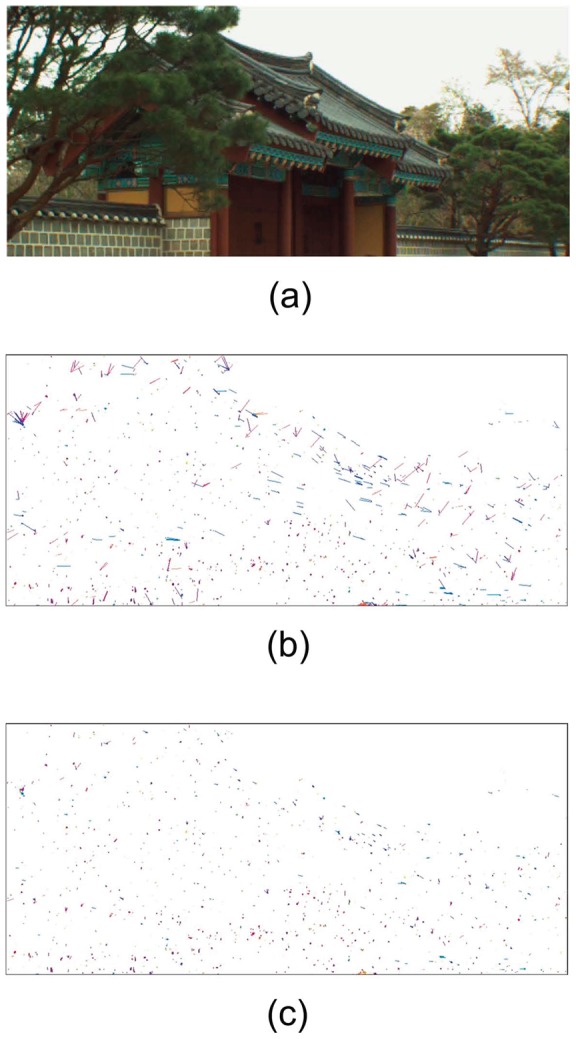
Result of rectification on optical flow field. (a) The almost static scene. (b) The obtained optical flow field without rectification. (c) The rectified optical flow field.



Based on 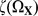 and 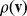, we can know the status of pixel 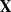 and its surroundings, and make appropriate rectifications and operations on them. There are several cases for different combinations of decisions caused by the binary value of 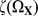 and 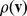. For the first case, when the pixel 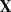 is occluded by other objects (i.e., 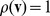), the vector 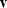 for 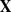 is an error PMV since actually no corresponding pixel can be found for 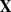, no matter 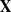 is surrounded by textures or not. In this case, it is not an easy way to predict a proper value for 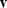 directly from neighboring vectors. Therefore, we mark 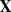 with a label 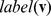, treat 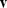 as unreliable and process the depth value for 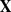 after the depth map has been reconstructed. Then for the second case, the pixel 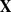 is visible and surrounded by textures (i.e., 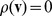 and 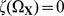). Texture information is benefit for accurate optical flow calculation, and thus the vector 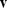 can be treated as reliable and accurate. Finally, the pixel 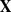 is visible but in a low textural region (i.e., 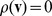 and 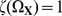). Low textural region can cause pixel-wise ambiguous vectors in optical flow calculation. These unreliable PMVs of ambiguous are usually odd when comparing with neighboring vectors, as can be found in Figure 3(a). In this case, the unreliable odd vectors can be processed by an average filtering with the neighboring vectors. We summarize the above processing as a condition function

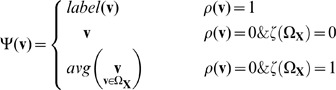
(4)where 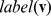 is a mark on 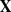 that 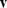 is reserved for the next step processing, 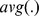 is an average operator on a set.

Based on the above occlusive and low textural region detection and rectification, a part of the obtained unreliable (i.e., odd) PMVs can be rectified effectively, and the error PMVs from occlusive regions are reserved for later processing. Therefore, the accuracy of PMVs is improved.

### Depth Map Reconstruction

Figure 3 provides the results of occlusive and low textural region detection and rectification. The results are obtained from two consequent color images in “Lovebird1” from MPEG [24]. The optical flow filed is obtained between 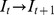 (i.e., from the previous image to the current one), and errors will occur in low textural regions in background and occlusions around object boundaries. Figure 3(a) shows the static scene with occlusive and low textural regions, and Figure 3(b) is the obtained optical flow field based on the given static scene of Figure 3(a) where many unreliable PMVs can be found. Figure 3(c) shows the result that Figure 3(b) processed by the operator in Equation 4. It can be found that most of the odd PMVs have been rectified.

After that, the vacant depth map 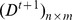 at time 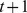 can be propagated and reconstructed via the obtained optical flow field 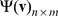 from the previous depth map as
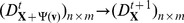
(5)


A depth map sequence that synchronized with the color frames can be reconstructed by Equation 5. This depth map sequence is with high resolution in video-rate. The depth information in vacant time slot is propagated from the key depth map, where the depth information is reliable. However, the processing on the reconstructed depth maps is not finish yet. As denoted by Equation 4, the PMVs for occlusive region is labeled and reserved for post-processing, and the regions that reserved will be a hole without depth information in the reconstructed depth map. On the other hand, ambiguous PMVs is inevitable in optical flow algorithms. These PMVs also can result in holes. Therefore, a depth map filtering for post-processing is necessary to improve the quality of reconstructed depth maps.

### Depth Map Filtering

As mentioned above, the reconstructed depth maps may contain holes due to the labeling operation in Equation 4, and ambiguous PMVs. For the ambiguous PMVs on pixel 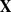, the missing depth value 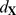 is very close to its spatial neighbors 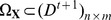, so that a median filter is applied. The operation can be denoted by
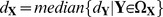
(6)


For the hole caused occlusions and marked in Equation 4, the pixel 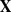 is marked by 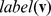. In this case, the missing depth value 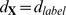 on pixel 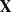 can be joint predicted by the depth value around the hole and the region where the PMV pointing to. We propose a depth value predictor as
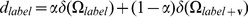
(7)where 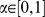 is normalized by the norm of 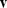, 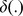 is an in-painting operator [25].

However, in-painting on depth map will bring noticeable blur effect, especially when the hole crosses the boundaries of high contrast edges. Usually, depth map with blurred boundaries results in a failure on foreground-background separation [26]. Therefore, an object boundary enhancement filter (BEF) is further proposed
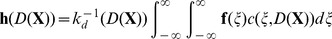
(8)where



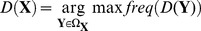
(9)is the depth value that most frequent appearing in 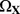, 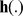 is a bilateral filter defined in [27]. 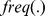 is a statistical function that count the appearing frequency of each element in a data set. For example, suppose we have a data set 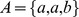, the result of 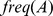 will be 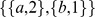. Therefore, we can further have 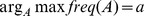.

**Table 1:** Challenges in dynamic 3D scene materials.

Sequence Name	Challenge
Dancer	Full tiny but orderless textures: miss-matches in motion estimation.
Balloon	Illumination variation and focus-light rotation: non-zero MV for static region.
Lovebird1	Zooming out movement: overlap of neighboring MVs.

Equations 8 and 9 can smooth the depth map, and the object boundary can be sharper.

**Figure 4:**
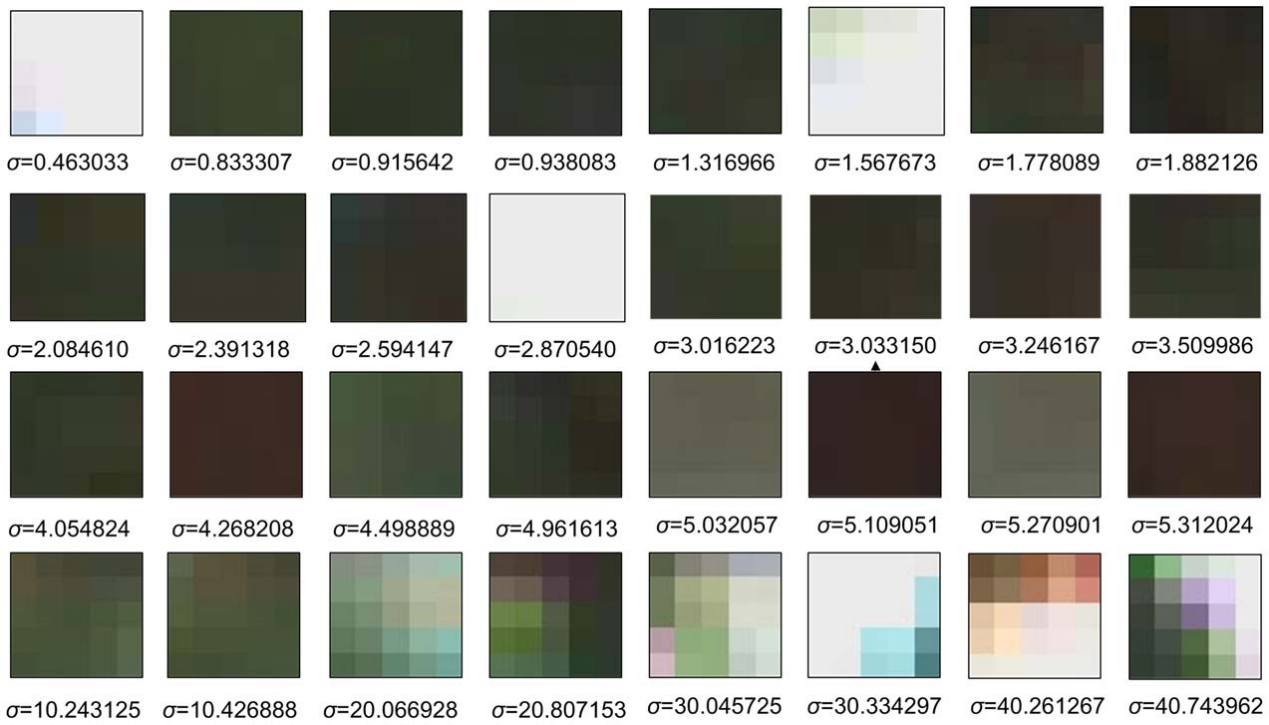
Texture analysis for color image for different 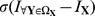.

### Dynamic 3D Scenes Materials

Dynamic 3D scenes contain a video-rate color image sequence that record the motion, color and texture information of this scene. Furthermore, a high resolution depth map sequence is also captured to record the 3D space information for all visible objects. As we have mentioned above, high resolution depth map cannot be captured by RGB-D or ToF cameras in video-rate that synchronically with the color image sequence so far. Recently, MPEG released their standard test sequences for dynamic 3D scenes with high resolution (more than standard definition) and high frame-rate, including color images and depth maps [24]. The color images were captured by cameras, but the depth maps were not captured but calculated by stereo matching and even manual labeling. The quality of depth map obtained through this way was assumed with the best quality to be obtained.

**Figure 5:**
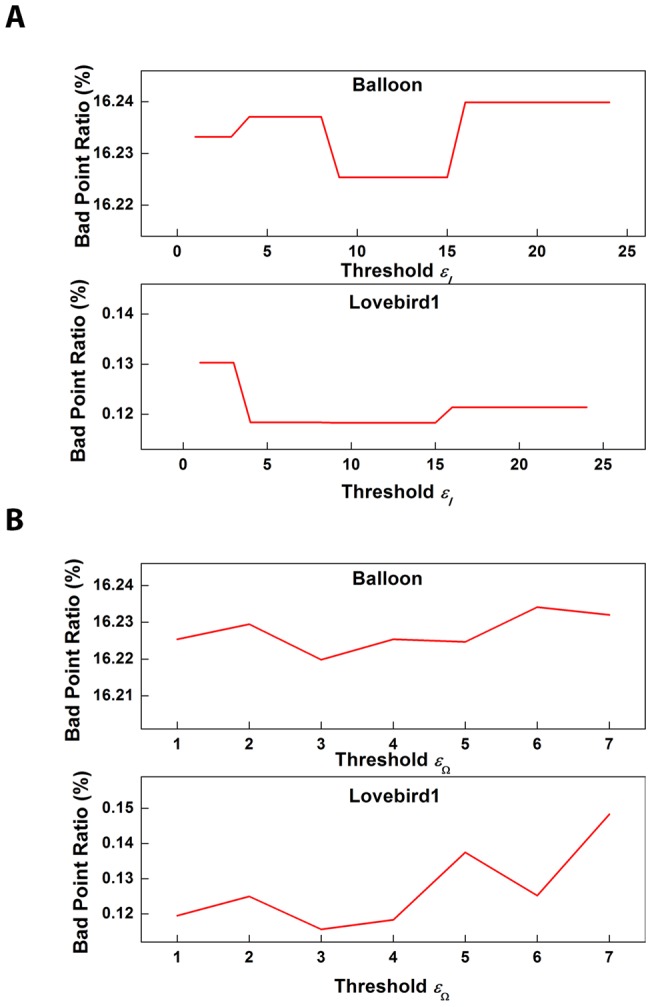
Parameter analysis for 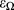 and 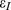. (a) The Bad Point Ratio with different 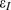 selection when 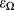 is fixed as 4. (b) The Bad Point Ratio with different 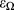 selection when 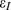 is fixed as 9.

The dynamic 3D scene materials named as Undo Dancer, Lovebird1, and Balloon will be used to testify our proposed algorithm. The captured color images and calculated depth maps of these materials are selected from [24]. These materials are with different challenges in depth reconstruction, as listed in Table 1.

**Figure 6:**
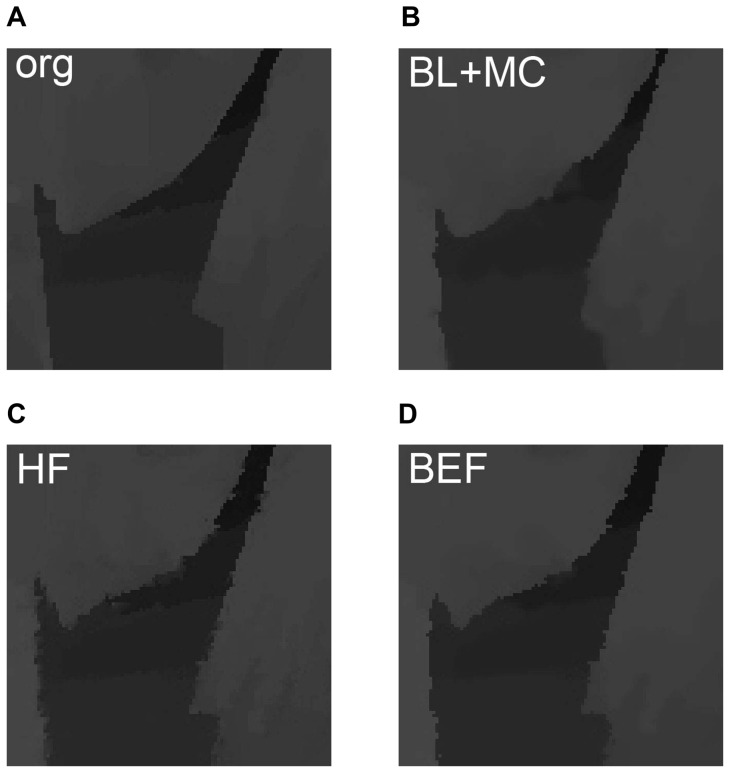
Subjective results on depth images for different filters. (a) Original depth map to be reconstructed. (b) Result of method in [19]. (c) Obtained depth map with hole-filling but without boundary enhancement filter. (d) Result of the proposed method.

**Figure 7:**
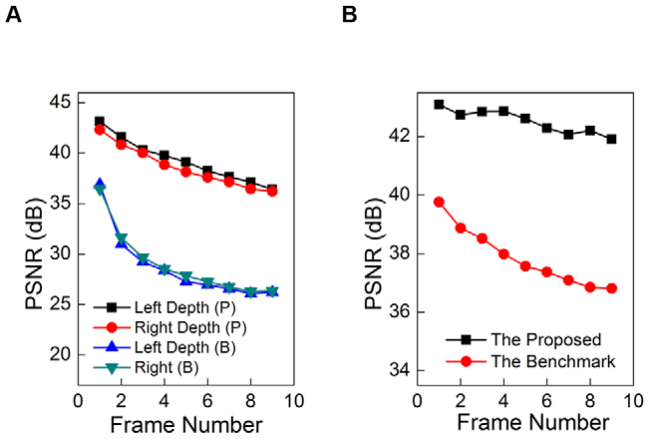
Objective results of our method. (a) Reconstructed quality for 9 consequent depth maps of left and right views for Lovebird1. (b) Quality for 9 synthesized correspondent virtual view images.

**Table 2:** Comparisons on Quality of Reconstructed Depth Maps and Synthesized Virtual Views (dB).

Test	Scheme	Left	Right	Virtual	Gain in	Gain in	Gain in
Sequence		View	View	View	Left View	Right View	Virtual View
Dancer	Proposed	**31.7396**	**31.7792**	**37.8609**	5.7873	5.7809	0.7355
	[19]	25.9523	25.9983	37.1254			
Lovebird1	Proposed	**39.2648**	**38.6206**	**41.8520**	10.5336	9.6364	3.9791
	[19]	28.7312	28.9842	37.8729			
Balloon	Proposed	**32.4155**	**31.0498**	**42.5151**	8.6305	7.9726	4.5184
	[19]	23.7850	23.0772	37.9967			

## Results and Discussion

### Experiment Arrangements

Experiments are arranged in four parts, including a discussion on thresholds in Equations 2 and 3, subjective and objective quality comparisons on depth reconstruction between our algorithm and the benchmark state-of-the-art method in [19], and finally an objective quality comparison on the dynamic 3D scene representation. In [19], the PMVs between consequent color images are not processed before propagation. Instead, a bilateral filter was applied for propagation, and errors in reconstructed depth were processed by motion compensation.

In our experiment, the depth map at 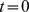 is selected from the given depth map sequence and treated as key depth map. The consequent depth maps in material are treated as vacant, and they will be propagated and reconstructed by our proposed algorithm and the benchmark method with the help of the key depth map. The consequent depth maps in material will be used as anchor for the reconstructed depth in objective quality evaluation.

### On the Parameters 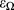 and 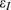


From Equations 2 and 3 we can see that there are two thresholds 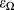 and 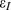 in our formulation. They modulate the number of the pixels of occluded or low textural, and thus the final quality of output propagated depth maps. These parameters (i.e., thresholds) are usually used in pixel classification. For parameter 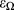, it is a real number varies in 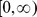, and it determines the number of low textural pixels. If 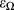 tends to be infinite, all pixels in image will be determined as low textural ones no matter how many textures around them. According to our proposed scheme, spatial filter (i.e., average filtering in Equation 4) is applied on the low textural pixels. Depth information for textural pixels can then be erased by this filter, and thus the accuracy of obtained depth map will be degraded. For parameter 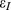, it is also a real number varies in 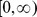, and it determines the number of occluded pixels. Furthermore, Equation 3 is performed on two corresponding pixels that related by vector 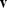. The accuracy of 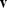 can be represented by the deference of 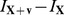 and checked by 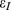.

Figure 4 demonstrates a texture analysis of 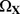 on one color image in test material Lovebird1 when 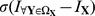 is changing. Texture in image can be treated as wave variation in signal. According to the definition of information entropy, more information is contained in 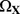 when the signal varying sharply. When considering the matching operation in optical flow calculation, more information in 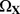 is helpful to obtain higher accurate and reliable 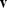. Therefore, the parameter of 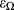 is also a threshold to distinguish reliable and unreliable 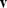. Figure 4 shows that the region 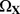 can be clearly classified to low textural region when 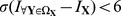, or otherwise, apparent textures are visible in 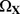.

Based on the texture analysis in Figure 4, the parameter settings for 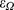 and 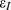 can be solved. Figure 5 demonstrate the performance curves (i.e., Bad Point Ratio) with respect to the variation of 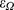 and 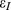. In Figure 5 (a), we fix 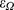 to be 4 and vary 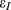 from 1 to 20. We can see that Bad Point Ratio drops to minimum point when 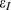 is 9. After that, in Figure 5 (b), we fix 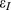 to be 9 and vary 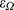 from 1 to 7. It can be found that Bad Point Ratio varies slightly for the parameter 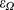, but the curve is increasing when 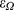 becomes larger. Therefore, we select 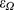 to be 3 to obtain a relative smaller Bad Point Ratio, indicating higher accuracy of depth map.

### Subjective Results for Depth Reconstruction

Figure 6 gives comparison results of subjective experiments. Each subfigure provides an enlarged part, and details the difference between our algorithm and the method in [19]. Figure 6(a) is the original depth map that selected in materials, and it serves as benchmark and is treated as absent in depth reconstruction. Figure 6(b) marked by “BL+MC” is obtained by method in [19], and it shows definite geometric distortion around the regions of moving object boundary. The phenomenon is a result of temporal bilateral-filtering. On the contrary, our algorithm detects the occlusive and low textural region, and processes these regions according to their types before depth propagation and reconstruction. Figure 6(c) marked by “HF” is the reconstructed depth map by using the optical flow field 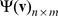 in Figure 3(c) with Equations 5, 6 and 7. As we mentioned above, the blurring effect is occurred around object boundaries. Figure 6(d) marked by “BEF” is the result obtained by our proposed method. The operation difference between Figures 3(c) and (d) is the BEF, which is processed by Equation 8 and 9. It can be found that the blurring effect is removed and the boundary around object is sharper.

### Objective Results for Depth Reconstruction

The objective quality comparison is measured by the peak signal-noise ratio (PSNR) from the reconstructed and corresponding existing depth maps from test materials. In the comparisons, higher PSNR indicates higher accuracy and better performance. Figure 7 and Table 2 provide the quantitative results. We can see that high precision of depth propagation is benefit in high quality of depth reconstruction, and the quality of reconstructed depth map of our method (labeled with “P”) is more than 8 dB better than the benchmark (labeled with “B”). However, errors (e.g. distortions around boundary) will also be propagated as shown in Figure 6(b). Therefore, the quality of reconstructed depth map will drop down along with longer distance propagation. As for the results given in Figure 7(a), we reconstruct 9 consequent depth maps for Lovebird1 for both left and right views respectively. Figure 7(a) shows that the quality of the 1st depth map reconstructed by the benchmark is comparable with that of the 9th by our method, indicates the higher quality of our method. On the other hand, Table 2 lists the average quality results on 9 reconstructed depth maps for three test sequences. It is obvious that our method has at least 5 dB gains on depth reconstruction, which is due to the rectification on optical flow field. On the other hand, BEF is used to eliminate the blur effect around boundary, and it will also benefit the quality.

### Results in Dynamic 3D Scene Representation

Dynamic 3D scene representation is measured by the objective quality of virtual view synthesis. Virtual view is an important application in 3D computer vision when color images and the corresponding depth maps are both available for a dynamic 3D scene [13], [17]. Better quality of depth maps can yield high quality of virtual view, and have better performance in dynamic 3D scene representation.

We use the reconstructed depth map for synthesis by VSRS software [28], which is a common test platform. The results are also given in Figure 7(b) and Table 2. Our method achieves 0.7 to 4.5 dB gains on PSNR for all the test materials. On the other hand, the accuracy of reconstructed depth map from the benchmark will be greatly affected by filter-based propagation. The distortion results in synthesis distortions.

### Summary of Results

In sum of the above quantitative comparisons, the proposed algorithm can achieve more accurate depth reconstruction on all test sequences with different challenges, including global motion and local motion, or dynamic scene that captured in natural environment and generated by computer graphics.
